# S100A4 (Mts1) gene overexpression is associated with invasion and metastasis of papillary thyroid carcinoma

**DOI:** 10.1038/sj.bjc.6602856

**Published:** 2005-11-01

**Authors:** M Zou, R S Al-Baradie, H Al-Hindi, N R Farid, Y Shi

**Affiliations:** 1 Department of Genetics, King Faisal Specialist Hospital and Research Center, PO Box 3354, Riyadh 11211, Saudi Arabia; 2 Department of Biological and Medical Research, King Faisal Specialist Hospital and Research Center, PO Box 3354, Riyadh 11211, Saudi Arabia; 3 Department of Pathology, King Faisal Specialist Hospital and Research Center, PO Box 3354, Riyadh 11211, Saudi Arabia; 4 Osancor Biotech Inc., Watford, Herts WD17 3BY, UK

**Keywords:** gene expression, thyroid neoplasm, metastasis, mts1 protein, S100A4

## Abstract

Tumour cell invasion and metastasis are the hallmark of malignant neoplasm. S100A4 is a member of small calcium-binding protein family and is involved in the cell proliferation and cancer progression. S100A4 is capable of inducing metastasis in animal models and is associated with aggressive phenotype of human tumours. We previously identified S100A4 as a candidate gene involved in anaplastic thyroid cancer metastasis by microarray analysis. To further determine whether S100A4 overexpression is associated with thyroid tumour invasion and metastasis, in the present study, we examined S100A4 gene expression in six benign multinodular goitres (MNG) and 28 matched samples of adjacent normal thyroid tissue (N), primary (T) and metastatic (M) papillary thyroid carcinomas (PTC) by immunohistochemistry and real-time reverse transcription–polymerase chain reaction (RT-PCR) analysis. This gave us the advantage of directly comparing levels of S100A4 expression within the same genetic background. Using immunohistochemistry, we found that high levels of S100A4 were detected in 24 of 28 (86%) PTC specimens and their local regional lymph node or distant metastases. No S100A4 staining was observed in normal thyroid tissues and simple MNG. However, in MNG coexistent with PTC, moderate focal staining could be found in 11 of 15 MNG adjacent to PTC. The S100A4 was stained more intensely in invading fronts than in central portions of both T and M. Real-time RT–PCR analysis of primary tumours and their matched lymph node metastasis demonstrated that significantly higher S100A4 transcripts were present in metastatic tumours as compared to the primary tumours (*P*<0.01). These data suggest that overexpression of S100A4 is associated with thyroid tumour invasion and metastasis and it may be a potential target for therapeutic intervention.

Thyroid cancers of follicular cell origin are the most common endocrine malignancies (Kinder, 2003; Sherman, 2003). Although the survival rate of patients with well-differentiated thyroid cancer exceeds the rate for most other cancers, the development of metastasis continues to be the most significant cause in thyroid cancer mortality (Mazzaferri, 1999; Sherman, 2003). Multiple pathogenic steps are involved in tumour invasion and metastases. These include the proliferation and detachment of tumour cells from the primary neoplasm, invasion of the surrounding extracellular matrix, angiogenesis, vascular or lymphatic dissemination, and eventually, homing of the tumour cells and proliferation at the new sites (Woodhouse *et al*, 1997; Hanahan and Weinberg, 2000; Liotta and Kohn, 2001). Identification of genes involved in this process will enable us to target them for future diagnosis or therapeutic intervention.

The S100A4 has recently emerged as an important protein with the capacity to promote invasion and metastasis of many human neoplasms (Barraclough, 1998; Mazzucchelli, 2002; Taylor *et al*, 2002; Cho *et al*, 2003; Lee *et al*, 2004; Moriyama-Kita *et al*, 2004). The human S100A4 gene, located on 1q21(Schafer *et al*, 1995), encodes an acidic 101 amino-acid protein with two EF-hand motifs. These motifs consist of a consensus sequence of 12 amino acids capable of binding Ca^2+^ with high affinity and specificity (Donato, 2001; Heizmann *et al*, 2002). S100A4 is member of S100 calcium-binding proteins that regulate intracellular processes such as cell growth, motility, cell cycle, transcription and differentiation. In all, 20 members of S100 protein family have been identified so far, and altogether, S100 proteins represent the largest subgroup in the EF-hand Ca^2+^-binding protein family (Heizmann *et al*, 2002). S100 proteins have received increasing attention due to their close association with several human diseases including cardiomyopathy (Most *et al*, 2001, 2003; Du *et al*, 2002), neurodegenerative disorders (Heizmann, 1999; Hoyaux *et al*, 2000), neuroprotection (Ahlemeyer *et al*, 2000; Pedersen *et al*, 2004), and cancer (Lee *et al*, 2004; Lin *et al*, 2004). These proteins have also been shown to be valuable markers in the diagnosis and management of these diseases and are considered to have a potential as drug targets to improve therapies (Kiewitz *et al*, 2000; Diederichs *et al*, 2004; Harpio and Einarsson, 2004; Rehman *et al*, 2004).

We have previously demonstrated by microarray analysis that S100A4 was highly expressed in anaplastic thyroid carcinoma cell line with high metastatic potential (ARO/met2) as compared to its parental cell line ARO and found that S100A4 overexpression was associated with advanced disease stage (Zou *et al*, 2004). Presently, little is known about the potential role of S100A4 in thyroid tumour invasion and metastasis. To this end, we examined S100A4 gene expression in 6 benign multinodular goitres (MNG) and 28 matched samples of adjacent normal thyroid tissue (N), primary (T) and metastatic (M) papillary thyroid carcinomas (PTC) by immunohistochemistry and real-time RT–PCR analysis. The results suggest that overexpression of S100A4 may play an important role in thyroid tumour invasion and metastasis.

## MATERIALS AND METHODS

### Thyroid tumour specimens

Fresh human tumour tissues with matched normal thyroid tissue and lymph node metastasis were obtained from 10 patients at surgery, and were immediately frozen in liquid nitrogen and stored at −70°C until processed. A pathologist confirmed that tissues from lymph node metastases were comprised by at least 80% of tumour tissue. A series of six benign MNG, 28 PTC with matched local regional lymph node or distant metastases (25 with lymph node metastasis and three with nose, lung, or brain distant metastasis, respectively) were selected solely on the basis of tissue availability for immunohistochemistry analysis. Among the 28 PTC samples, 24 are classic PTC, one tall cell variant and two follicular variant of PTC. The institutional review board approved the research project.

### Immunohistochemistry analysis

A representative formalin-fixed paraffin-embedded tissue block was chosen from the pathology archives for each of the 28 PTC and six MNG selected for immunostaining. Normal thyroid tissues were also contained in these tissue blocks. Sections (4 *μ*m) mounted on poly-L-lysine-coated slide were incubated for 30 min at 60°C, deparaffinised by standard methods, and placed in 0.05 M Tris-HCI buffer, pH 7.2. Antigen retrieval was performed for 20 min in 10 mm sodium citrate buffer (pH 6) heated at 95°C in a steamer, followed by cooling for 20 min. After blocking endogenous peroxidase activity with 0.3% aqueous hydrogen peroxide for 5 min, the primary polyclonal rabbit anti-S100A4 antibody (DAKO, Carpinteria, CA, USA) was incubated with the sections at a final dilution of 2 *μ*g ml^−1^ for 30 min. In immunobloting analysis using recombinant S100A1, S100A2, S100A4, S100A6, and S100B, the antibody recognised only S100A4. No crossreaction with the other S100 proteins was observed. For each case, a control slide was incubated with Tris-HCI buffer substituted for the primary antibody. DAKO LSAB+kit, HRP was used for the detection of the immunostaining. The sections were counterstained with Mayer's haematoxylin. The distribution of immunolabelling was determined from a minimum of three representative high-power (× 400) fields and categorised into three groups: 0%, negative; 1–25%, focal; and 26–100%, diffuse.

### Quantitative real-time RT–PCR analysis

Total RNAs from normal thyroid, thyroid tumour, and lymph node metastasis were extracted by quanidinium thiocyanate–phenol–chloroform method as described previously (Shi *et al*, 1999). The integrity of RNA was verified by denaturing gel electrophoresis. Of total RNA, 2 *μ*g were reverse-transcribed using Promega reverse transcription system (Promega, Madison, WI, USA). LightCycler DNA Master SYBR Green 1kit was used for quantitative real-time PCR analysis according to the manufacturer's protocols (Roche, Mannheim, Germany). The cDNA mix was diluted 10-fold and 2 *μ*l of the dilution were used for real-time PCR analysis. PCR primers for 440 bp S100A4 cDNA fragment were 5′-TCTTTCTTGGTTTGATCCTG-3′ (sense) and 5′-GCATCAAGCACGTGTCTGAA-3′ (antisense). The sense primer spans over the intron 1(938 bp) so that the contaminating genomic DNA would not be amplified as the expected 440 bp cDNA fragment. The mRNA level of housekeeping gene glyceraldehydes-3-phosphate dehydrogenase (GAPDH) was used as an internal control and a 300 bp PCR product was amplified using the following two primers: 5′-ACAGTCAGCCGCATCTTCTT-3′ (sense) and 5′-TTGATTTTGGAGGGATCTCG-3′ (antisense). The PCR conditions are 95°C for 30 s followed by 40 cycles of amplification (95°C for 10 s, 48°C for 5 s, and 72°C for 10 s). The resulting concentration of S100A4 PCR products were normalised by comparison with GAPDH and was used to determine the mRNA level of S100A4 among thyroid tumour specimens.

### Statistical analysis

The significant difference of S100A4 gene expression between specimen groups was carried out using the unpaired Student's *t*-test. Differences were considered statistically significant when the *P*-value was <0.05.

## RESULTS

### Immunohistochemical analysis of S100A4 expression in papillary thyroid cancer specimens

**Figure 1:**
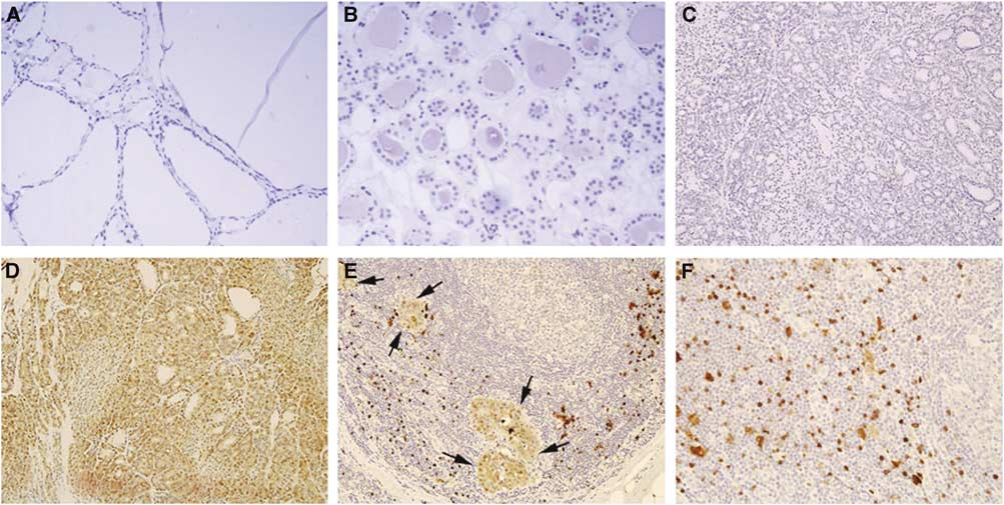
S100A4 immunostaining of tissues from normal thyroid, benign multinodular goitre, primary papillary thyroid carcinoma, and its lymph node metastasis. Negative staining in both normal thyroid (**A**, × 100) and multinodular goitre (**B**, × 100); negative staining of papillary thyroid carcinoma with a secondary anti-rabbit antibody only (**C**, × 100) and positive staining of the same tissue when S100A4 antibody was added (**D**, × 100); focal lymph node metastasis of papillary thyroid carcinoma stained with S100A4 (**E**, × 100). An arrow indicated the metastatic foci. Strong staining of some lymphocytes, dendritic, and stromal cells were also shown surrounding the germinal centre, which is not stained (**E**, × 100; **F**, × 200).

**Figure 2:**
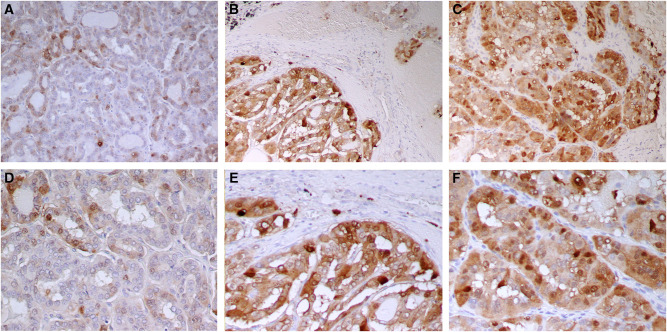
Immunostaining of primary and metastatic papillary thyroid carcinoma cells with anti-S100A4 polyclonal antibody from the same patient. Weak cytoplasmic labelling of papillary thyroid carcinoma cells in the central portion of the tumour at the magnifications × 100 (**A**) and × 200 (**D**). Strong cytoplasmic labelling of carcinoma cells at the invading front of primary tumour × 100 (**B**) and × 200 (**E**), and in metastatic lymph node at the magnifications of × 100 (**C**) and × 200 (**F**).

**Figure 3:**
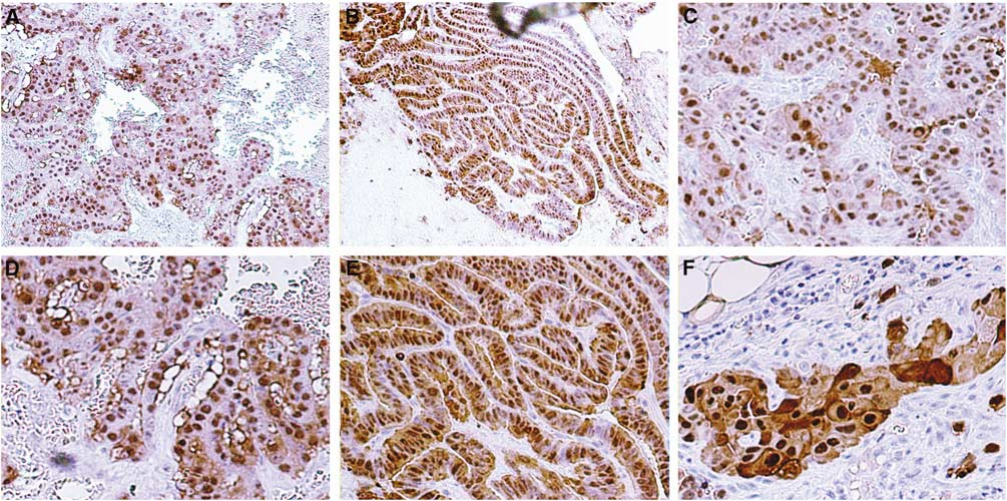
Nuclear staining of papillary thyroid carcinoma cells with anti-S100A4 polyclonal antibody. Weak cytoplasmic and strong nuclear labelling of primary PTC (**A**, × 100; **D**, × 200) in a patient who developed distant metastasis (carcinoma cells metastasised to nose) 8 years after surgical removal of the primary thyroid tumour. Stronger cytoplasmic and nuclear labelling of the metastatic PTC is shown from the same patient (**B**, × 100; **E**, × 200). Weak cytoplasmic and strong nuclear labelling of primary PTC is shown in another patient (**C**, × 200). Stronger cytoplasmic and nuclear labelling of papillary thyroid carcinoma cells that metastasised to a regional lymph node of the same patient who later developed lung metastasis 1 year after treatment (**F**, × 200).

**Figure 4:**
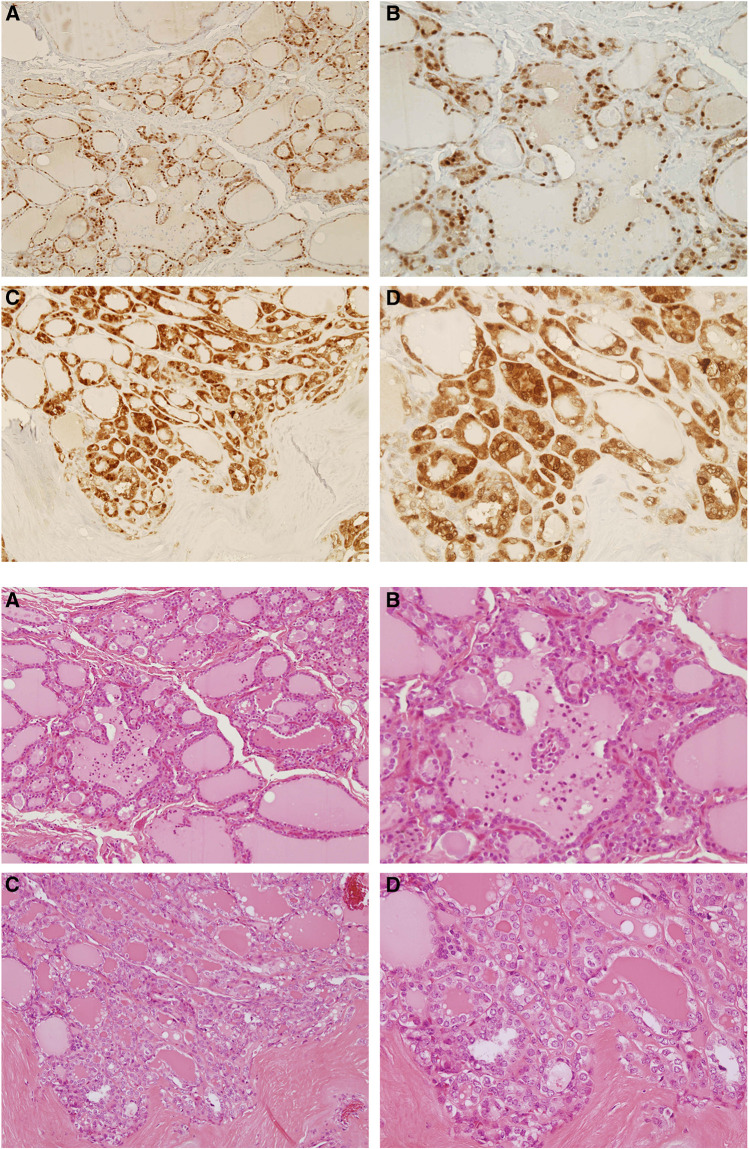
Focal S100A4 immunostaining of thyroid follicular cells in a multinodular goitre coexisted with papillary thyroid carcinoma. Moderate focal cytoplasmic and nuclear labelling of thyroid follicular cells can be seen in a multinodular goitre (upper panel: **A**, × 100; **B**, × 200), which is adjacent to papillary thyroid carcinoma. Strong labelling was shown in thyroid papillary carcinoma cells at the invading front from the same patient (upper panel: **C**, × 100; **D**, × 200). The lower panel shows the conventional haematoxylin and eosin staining of the corresponding areas shown in the upper panel.

We demonstrated previously the S100A4 overexpression in advanced stage of thyroid carcinomas by Northern blot and real-time RT–PCR analysis. To examine the expression of S100A4 at the cellular level, we performed immunohistochemical analyses using histochemical preparations of formalin-fixed paraffin-embedded surgical specimens. Normal thyroid follicular cells were not stained with the S100A4 antibody (Figure 1A). No immunostaining was found in samples incubated with the secondary antibodies in the absence of primary antibodies (Figure 1C). Strong immunostaining was present in some lymphocytes, dendritic cells, and fibroblast-like reactive stroma cells in the thyroid tissues and lymph nodes usually surrounding the germinal centres (Figure 1E and F), which served as an internal positive control as previously reported (Rudland *et al*, 2000; Rosty *et al*, 2002). As shown in Table 1, S100A4 immunostaining was detected in 24 out of 28 (86%) PTC specimens: 21 with diffuse staining (staining was present in more than 25% of tumour cells) and three with focal staining (staining was present in <25% of tumour cells). In these S100A4-positive PTC samples, stronger staining was often observed at the tumour-invading front (Figure 2B and E) as compared to the central region (Figure 2A and D). In general, metastatic PTC samples had stronger staining (Figure 2C and F) when compared with their matched primary tumours (Figure 2A and D). There are two cases where positive staining was found in the metastatic lymph nodes and negative staining in the primary tumours. Therefore, 26 metastatic PTCs were S100A4 positive. We next compared the intensity of S100A4 staining between primary and metastatic PTCs. In all, 17 out of 24 (71%) metastatic PTCs were found to have stronger immunostaining than their matched primary PTC samples: 14 with local regional lymph node metastasis and three with distant metastases (one metastasised to the nose 8 years after treatment, and the other two metastasised to the lung and brain frontal lobe, respectively at the time of diagnosis) (Figure 3). Equal intensity of staining was found in the remaining seven metastatic tumours as compared to the primary tumours. S100A4 staining was mainly cytoplasmic, heterogeneous in some tumours with tendency to more intense staining in invading fronts than in central portions of the tumour samples. Striking nuclear staining was found in five tumour samples: three with distant metastasis to nose, lung or brain frontal lobe (TNM stage IV), and the other two with stage III tumours, indicating that nuclear staining may be associated with aggressive behaviour of the cancer (Figure 3). Among the four primary PTC samples with negative S100A4 staining, strong staining was observed in two matched lymph node metastasis tumour tissues. Interestingly, in 11 out of 15 cases where MNG were next to PTC, focal moderate cytoplasmic and nuclear staining could be found in MNG (Figure 4, upper panels A and B) as compared to the stronger staining in PTC (Figure 4, upper panels C and D). Examination of the same MNG area stained by haematoxylin and eosin failed to show the diagnostic nuclear features of PTC such as clearing of the nucleoplasm, peripheral margination of chromatin, or nuclear grooves, although subtle nuclear atypia manifesting as nucleomegaly and slight irregularity of the nuclear membrane was evident (Figure 4, lower panels A and B). These features were, however, present in the PTC area of the same patient (Figure 4, lower panels C and D). However, in the six cases with simple MNG, none of them labelled by S100A4 (Figure 1B).

### Analysis of thyroid tumour specimens for S100A4 gene expression by quantitative real-time RT–PCR analysis

**Figure 5:**
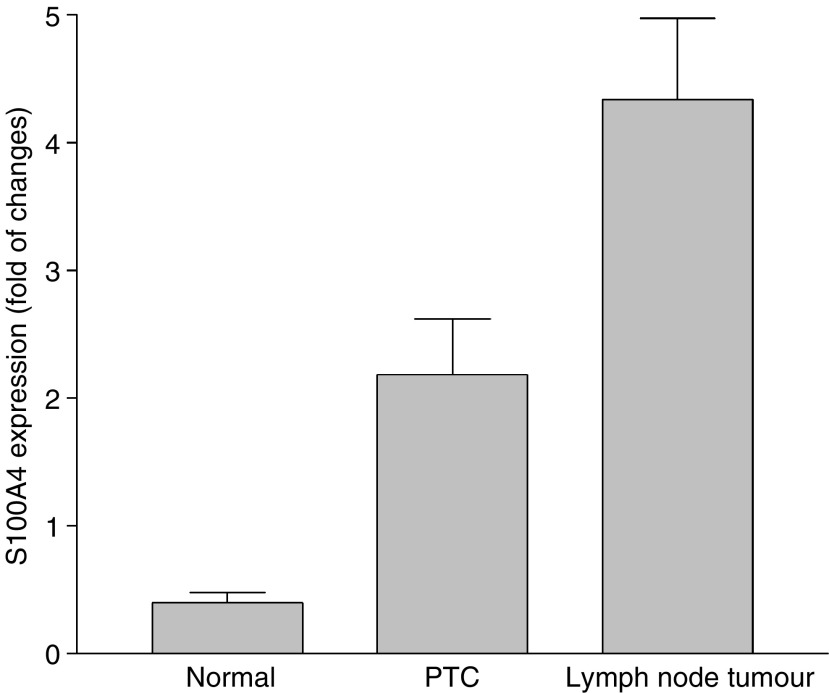
Comparison of S100A4 expression in tissues from normal thyroid, papillary thyroid carcinoma, and its lymph node metastasis by real-time quantitative RT–PCR. Total RNAs were isolated from 10 patients with matched tissues of normal thyroid, papillary thyroid carcinoma, and its lymph node metastasis. After reverse transcription, S100A4 mRNA levels among different tissues were determined by quantitative real-time PCR. The fold of changes in S100A4 expression among 10 matched tissue samples (means±s.e.m. of three separate experiments) was shown after normalisation to that of GAPDH expression.

Since S100A4 was intensely immunostained in metastatic tumours as compared to the primary tumours, we wondered whether its mRNA transcripts were also increased to the same degree as its protein expression. To this end, we analysed S100A4 transcripts from 10 patients with matched tissues of normal thyroid, PTC and lymph node metastasis by real-time RT–PCR. As shown in Figure 5, significantly higher S100A4 expression was seen in metastatic tumours as compared to primary tumours (*P*<0.01) even though S100A4 overexpression was present in primary tumours when compared with normal thyroid tissue (*P*<0.01). These data suggest that S100A4 overexpression was involved in tumour invasion and metastasis.

## DISCUSSION

S100A4 is known to be involved in the tumour invasion and metastasis by virtue of its ability to activate nonmuscle myosin (Ford *et al*, 1997; Takenaga *et al*, 1997a; Bjornland *et al*, 1999; Li *et al*, 2003; Jenkinson *et al*, 2004). However, information on S100A4 expression in thyroid tumour cells is limited thus far, although high S100A4 expression in breast, ovary, colon, gastric, and pancreatic carcinomas has been reported (Mazzucchelli, 2002). In an early study, we have shown increased S100A4 expression in thyroid carcinoma specimens with advanced disease stage and indicated that it may be a useful prognostic marker for thyroid carcinoma (Zou *et al*, 2004). In the present study, we have shown increased S100A4 expression in thyroid tumour cells metastasised to regional lymph nodes or distant organs and at the tumour invasion front in matched primary thyroid tumour and their metastatic specimens. These findings provide further support that S100A4 is involved in thyroid cancer invasion and metastasis.

Since S100A4 is expressed in a variety of cell types such as lymphocytes, dendritic cells, macrophages, and smooth muscles (Takenaga *et al*, 1997b; Rudland *et al*, 2000), it is important to examine S100A4 expression at the cellular level to ascertain that higher expression of S100A4 mRNA in thyroid carcinoma specimens reflects the expression in thyroid carcinoma cells themselves. To evaluate this point and examine more closely the S100A4 expression in invasive and metastatic cells, we performed immunohistochemical analysis using polyclonal rabbit anti-S100A4 antibody. Strong immunostaining was observed at the tumour invading front and metastatic site. This observation is further confirmed by quantitative real-time RT–PCR analysis, which shows higher S100A4 mRNA in lymph node metastasis as compared to primary tumours, supporting the role of S100A4 in cell motility and invasion (Ninomiya *et al*, 2001). Previous immunohistochemical studies of S100A4 showed an overexpression in 41% of breast carcinomas (Rudland *et al*, 2000), 55% of gastric carcinomas (Yonemura *et al*, 2000), 94% of colorectal adenocarcinomas (Takenaga *et al*, 1997b), 93% of invasive pancreatic carcinomas (Rosty *et al*, 2002), and 25% of oesophageal squamous carcinoma. Using tissue microarrays, Cross *et al* (2005) has recently demonstrated that S100A6, S100A8, S100A9, and S100A11 are all expressed in common cancers, especially breast cancer. Moreover, they found a translocation of S100A11 expression from exclusively nuclear location in normal tissues to cytoplasmic and nuclear in all common cancers. We instead found a translocation of S100A4 expression from cytoplasmic to nuclear in five samples with advanced disease stage, suggesting that S100A4 translocation to the nucleus may be related to the proliferation or metastatic potential of the cancer cells. Flatmark *et al* (2003) has demonstrated that S100A4 nuclear localisation correlated with tumour stage and aggressiveness of colorectal carcinoma. A recent study has shown that A100A1, another member of the S100 family of proteins, can interact with S100A4 to modulate the effect of S100A4 on their metastatic abilities (Wang *et al*, 2005).

Although it remained unclear what causes S100A4 overexpression in thyroid carcinomas, it has been shown that hypomethylation of S100A4 gene correlates with gene activation and overexpression in human pancreatic carcinomas and colon adenocarcinoma cell lines (Nakamura and Takenaga, 1998; Rosty *et al*, 2002). Hypomethylation of the first (Rosty *et al*, 2002) and second introns (Nakamura and Takenaga, 1998) were well correlated with the expression of S100A4. The addition of 5-Aza-2′-deoxycytidine, an inhibitor of the eukaryotic DNA methyltransferase, induced the expression of the S100A4 gene in colon adenocarcinoma cell lines (Nakamura and Takenaga, 1998). In humans, the gene for S100A4 occurs in a cluster of 13 S100 genes on chromosome 1q21 (Schafer *et al*, 1995; Heizmann *et al*, 2002). Gain of 1q and loss of 9q21.3–q32 have been reported to be associated with a less favourable prognosis in papillary thyroid carcinoma using comparative genomic hybridisation (Kjellman *et al*, 2001). Gain of 1q and loss of 9q21.3–q32 are exclusively seen in tumours from patients with aggressive disease, and the presence of distant metastases is associated with gain of 1q. It is possible that S100A4 may be one of those genes involved in the gain of 1q given that S100A4 is located in 1q21 region.

In the rodent model systems, elevated levels of S100A4 can only synergise with growth-promoting oncogenic products such as c-erbB-2/*neu* (Davies *et al*, 1996) or have to be expressed in benign tumours before metastasis can be induced (Lloyd *et al*, 1998). Transgenic mouse studies have demonstrated that S100A4 by itself is not able to initiate tumours or induce metastatic effect in normal rodent cells (Davies *et al*, 1995). However, it did induce metastatic disease of cells that had been initiated by oncogenes such as c-erbB-2/*neu*. When S100A4 transgenic mice were mated with *neu* transgenic mice, known for developing mammary cancer after multiple pregnancies, double-positive offspring that inherited both genes developed mammary tumours with significantly more lung metastases than mice that inherited only the *neu* oncogene (Davies *et al*, 1996). Overexpression of c-erbB-2/*neu* has been previously reported in PTC, and PTC without distant metastases showed significantly less cytoplasmic immunostaining than those with development of metastases (Haugen *et al*, 1992; Kremser *et al*, 2003). Taken together with our findings of S100A4 overexpression in advanced thyroid tumours, the transgenic mouse model may fit well with human PTC, implicating that S100A4 may enhance thyroid cancer invasion and metastasis in cooperation with c-erbB-2/*neu*. Interestingly, mice with a germline inactivation of the S100A4 gene have been found to develop spontaneous tumours as a result of destabilisation of the apoptosis-promoting function of p53 tumour suppressor gene. However, tumours developed in these mice were nonmetastatic (EL Naaman *et al*, 2004).

In contrast to the negative immunostaining in specimens of simple multinodular goitre, we detected focal immunostaining of MNG that was adjacent to papillary thyroid carcinoma. Although it is not clear what leads to the focal overexpression of S100A4, it may be resulted from local demethylation or hypomethylation, one of the early events in tumour development. Overexpression of S100A4 induced by this epigenetic event may contribute to abrogation of apoptosis and tumorigenesis (EL Naaman *et al*, 2004). It may also be an early sign of malignant transformation resulting from genetic mutations leading to oncogene activation and/or inactivation of tumour suppressor genes. This hypothesis was supported by transgenic mouse studies described above and previous studies demonstrating mutations of several oncogenes (ras, TRK, and gsp), tumour supperssor genes such as FHIT, and mitochondrial genes in benign MNG (Shi *et al*, 1991; Farid *et al*, 1995; Zou *et al*, 1999; Abu-Amero *et al*, 2005). Although these S100A4-positive cells lack characteristic morphological feature of PTC, we suggest that positive staining for S100A4 of a MNG should alert pathologist to the coexistence of PTC foci and should be verified in a large series of specimens.

In summary, we have investigated S100A4 expression at the molecular and cellular level in matched primary and metastatic PTC specimens. High levels of S100A4 were detected in 24 of 28 (86%) PTC specimens and their local regional lymph node or distant metastasis. The S100A4 expression was much higher at the tumour-invading front and in the metastatic tumours as compared to the primary tumours. These data suggest that overexpression of S100A4 is associated with thyroid tumour invasion and metastasis and it may be a potential target for therapeutic intervention.

## Figures and Tables

**Table 1:** S100A4 immunostaining in MNG and PTC from primary and metastatic regions

	**Simple MNG**	**MNG adjacent to PTC**	**Primary PTC**	**Metastatic PTC**
**S100A4 labelling**	**(*n*=6)**	**(*n*=15)**	**(*n*=28)**	**(*n*=28)**
Negative	6	4	4	2
Focal (1–25%)		11	3	2
Diffuse (26–100%)			21	24
